# Formal help for persons with multiple sclerosis—Background factors associated with usage of personal assistance and home help in Sweden

**DOI:** 10.1371/journal.pone.0286010

**Published:** 2023-05-18

**Authors:** Daniel Ståhl, Emilie Friberg

**Affiliations:** 1 Department of Social Work, University of Gothenburg, Gothenburg, Sweden; 2 Division of Insurance Medicine, Department of Clinical Neuroscience, Karolinska Institutet, Stockholm, Sweden; Faculty of Medicine, University of Belgrade, SERBIA

## Abstract

Multiple sclerosis (MS) is a chronic neurological disease that may cause several different symptoms, some which may entail the need for help in daily life. The aim of this study was to explore the association between sociodemographic background factors and the use of personal assistance and home help services (home help) among persons with MS in Sweden. The study was based on cross-sectional survey data merged with register data and included 3,863 persons with MS aged 20–51. Binary logistic regression analyses were performed to identify factors associated with the use of personal assistance and home help. The central finding of this study was that grade of impairment, as determined by the Expanded Disability Status Scale for Multiple Sclerosis (EDSS), was the most important variable associated with the use of both personal assistance (p < 0.001, OR 18.83) and home help (p < 0.001, OR 6.83). Living alone and receiving sickness benefit were also both associated with the use of personal assistance (p < 0.001, OR 3.32; p 0.001, OR 3.32) and home help (p 0.004, OR 2.56; p 0.011, OR 2.56). Stating a visible symptom of MS as being the most limiting factor of the disease (p 0.001, OR 2.73) and having a disposable income below the limit for poverty risk (p 0.02, OR 2.16) was associated with the use of personal assistance. Receiving informal, meaning unpaid, help (p 0.049, OR 1.89) was associated with the use of home help. Several background factors were controlled for but were not related to differences in the usage of formal help. The results indicated no significant differences in demographic characteristics that could be linked to unequal distribution. However, differences were found between those using personal assistance and home help. The latter were mainly affected by invisible symptoms, suggesting a plausible influencing factor in the chances of obtaining more comprehensive help in the form of personal assistance. Users of home help were also more likely to receive informal help than users of personal assistance, which may suggest that home help is not sufficient.

## Introduction

Multiple sclerosis (MS) is a chronic neurological disease that, unlike many other neurological conditions, affects persons from young adulthood. Between 2013 and 2020, the number of persons with MS worldwide increased from 2.3 million to 2.8 million [1]. Sweden is a country with a particularly high prevalence of persons with MS, approximately 189 per 100,000 [2]. MS can cause a range of symptoms, many of which are invisible to others. Examples of invisible symptoms related to MS are fatigue, cognitive impairment, and depression [3]. Previous research has shown that persons with MS who are classified by clinical assessment as being not highly affected by the disease still experience one or several invisible symptoms [4].

While it has been recognized that the prevalence of persons affected by impaired health and function will increase in the future, it has also been established that these persons experience societal barriers and discrimination, and have fewer social and economic opportunities than those without impaired health and/or function. It has also been recognized that this impact may differ based on demographic factors, such as sex, age, or ethnicity [5]. While Sweden extended the legal right to support and services for disabled people at the end of the 21st century, in line with the independent living movement, greater access is always balanced by the state urge to cut costs. Personal assistance, in particular, has been a subject of debate due to its cost and the alleged prevalence of benefit fraud [6–8]. Among the consequences of recent austerity measures, it has been noted that persons denied personal assistance are forced to rely on the relatively limited provisions of home help services (henceforth referred to as home help) or informal, unpaid help from family or friends [7].

*The aim of this study was to explore the association between sociodemographic background factors and the use of personal assistance or home help among persons with MS*.

### The Swedish context

Sweden is a country that provides universal social insurance, health care, and social services for its citizens, mainly financed by tax revenues. Help in daily living and activities is commonly divided between formal and informal help. Formal help implies that a user receives help from a paid employee. Informal help is defined as help provided by family, friends, and others who do not receive any payment [9, 10]. The focus of the present study is on personal assistance and home help, two forms of formal help for which the right to use is assessed on the basis of different legislation. Home help is provided according to the Social Services Act (SSA) and personal assistance is provided according to the Swedish Act concerning Support and Service for Persons with Certain Functional Impairments (LSS) [11]. It is imperative to note that the prerequisites for receiving help according to the SSA or LSS differ considerably. To be eligible for help under the SSA, a person must have some need of help in daily living, which can be caused by impairment and/or old age. Help under the SSA is intended to ensure a reasonable standard of living for the user [12]. The LSS, on the other hand, is a law that entitles those who receive help to good living conditions. To be eligible for help under the LSS, a person must have an impairment that is permanent and extensive. Furthermore, only persons under the age of 65 may be entitled to personal assistance [13].

### Literature review

Previous research derives from various national contexts. Some large-scale research has included comparisons between persons with MS and persons with other forms of chronic disease. In comparison, persons with MS were found to be one of four groups that used both formal and informal help the most [10]. A study specifically focusing on home help found that persons with MS were one of the youngest groups (mean age 59), had the largest proportion of females (70.7%), and had used home help for comparatively long periods of time (mean 3.5 years) compared to the other groups [14]. Another study showed that persons with MS more often reported insufficient help in certain daily activities and were twice as likely to report that they had no help or inadequate help compared to those with other diseases [15].

Studies specifically focusing on persons with MS have shown that use of informal help in general is more common than use of formal help. A similar patterns has been found in Sweden [16], the UK [17], and the US [18]. In Sweden, it was found that the use of home help (17%) and personal assistance (19%) occurred to about the same extent. However, the use of informal help was more common within the sample [16]. An older study from the UK found that 39% of those assessed as living with moderate impairment and 12% of those with severe impairment did not use help services, suggesting inadequate service provision [19].

Several studies have highlighted differences between subgroups of persons with MS. Women has been found to use more formal and informal help than men, although the odds ratios for men were larger regarding both formal and informal help [10]. Higher level of education has been shown to increase the likelihood of usage of personal assistance for a longer period of time [20]. Whether a person with MS lives alone or cohabitates has been shown to have a large impact on usage of help as informal help is mainly performed by family members [14, 18, 21]. Previous studies have suggested differences in service provision between urban and rural areas. However, service provision in these studies often includes both formal help and medical care [22, 23]. Severe impairment, mainly determined by a higher EDSS score (Expanded Disability Status Scale in Multiple Sclerosis), have been found to be related to higher use of formal and informal help [16, 20, 24, 25]. Living with MS can mean experiencing a number of invisible symptoms, such as fatigue. Johansson et al. [26] found differences between persons with MS depending on if they were affected by fatigue. For those not affected by fatigue, it was more common to use formal help, while the group affected by fatigue used informal help to a larger extent [26].

## Methodology

The present article is based on a cross-sectional survey on different aspects of life related to MS distributed in May 2021. All persons living in Sweden and listed in the Swedish MS Registry [27] between the ages of 20 and 50 were invited to participate in a web-based questionnaire administered by Statistics Sweden. Up to four reminders were sent to individuals who had not responded to the survey. In total, 8,458 persons were invited to participate. The response rate was 52% (4,412 participants). Informed consent was provided by all participants by sending in the survey. The study received ethical approval from the Swedish Ethical Review Authority (2020–04996).

### Data

The data used in this study derive from three sources. The survey requested information regarding several aspects of life. Respondents were asked whether they utilized formal help (e.g., personal assistance and home help) and/or informal help. Furthermore, the survey requested information on the symptom of MS that most affected the respondent’s daily life, if they had another long-term disease/impairment, their occupation, and if they were cohabitating, among other things. Data on sex, age, country of birth, level of education (number of years in school), type of residential area, and disposable income were obtained from Statistics Sweden’s Longitudinal Integrated Database for Health Insurance and Labor Market Studies (LISA) [28]. Data on the latest EDSS scores of the respondents were obtained from the Swedish MS Registry. In cases where the latest EDSS score was three years old or older, the data were considered as missing. Statistics Sweden linked the data sources using unique Swedish personal identification numbers and provided the data to the authors in anonymized form.

### Dependent and independent variables

Two binary categorical variables were used as dependent variables in separate multivariate logistic regression analyses: personal assistance (reference group no.) and home help (reference group no.).

Several independent variables were used in the models. Sex was included as a binary variable for the responses woman and man. The age variable was divided into three categories: ages 20–30, 31–40, and 41–51. The variable for country of birth was recoded into two groups: the Nordic countries, including Sweden, and other countries. Educational level was recoded as a binary variable, depending on if the respondent had studied for up to twelve years, corresponding to high school education in Sweden, or more. Disposable income was recoded as a binary variable: those with a disposable income over SEK 165,240 per year and those whose disposable income was less than this. This demarcation was made according to the measurement of poverty risk, corresponding to a disposable income below 60% of the median income of the country [29]. Data on disposable income were from 2019. The variable for type of residential area was based on three possible responses according to degree of urbanization: city, town or suburban area, or rural area [29]. The variable for occupation was binary, indicating if the respondent was working or receiving sickness benefit: sick leave compensation and/or disability pension. Both sick leave and disability pension are related to reduced work capacity due to disease/impairment and can be received at four different levels: 100%, 75%, 50%, or 25%. The respondents were asked to indicate the symptom of MS that most affected them: W*hich MS symptom do you find the most limiting*? As this was an open-ended question, responses were initially grouped based on categorizations from the UK MS clinical guideline on symptom management [30] and the American National MS Society [31], and then coded into groups: invisible symptom, visible symptom, or no symptom. Examples of symptoms coded as invisible include fatigue, sensory and pain symptoms, and bladder dysfunction. Examples of symptom coded as visible include movement problems, vision problems, and dysarthria. Respondents were also asked if they had another long-term disease or impairment, and responses were included as a dichotomous variable, indicating either absence or presence. The respondents were asked to indicate if they cohabited with others. They were also asked if they were receiving informal help. The variables for cohabitation and receiving informal help were both binary variables, corresponding to if the person lived in a single household or together with others. and if they received informal help. The variable for EDSS score was divided into three categories: 0–2.5, 3–5.5, and 6–9.5. A higher score on this measure indicates more severe impairment [32].

### Statistical analysis

Descriptive statistics were analyzed and presented for the following subgroups: those using personal assistance, those using home help, and those not using any formal help. Before testing the independent variables in the models, they were analyzed with the dependent variables via crosstabulation. Both Chi-Square tests and Fisher’s exact tests were performed, based on the assumption of goodness-of-fit tests of logistic regression [33]. Collinearity diagnostics were performed to control for potential multicollinearity between the independent variables. No multicollinearity was found. Several potential interaction effects were examined, for example, cohabitating or not and receiving informal help, receiving sickness benefits and the presence of another disease or impairment. No interaction effects were found. All independent variables were used as single predictors in a logistic regression analysis to obtain unadjusted odds ratios (OR) and associated p-values. In the next step, all independent variables were used simultaneously as predictors in a logistic regression analysis to obtain adjusted odds ratios (OR) and associated p-values. An adjusted odds ratio is controlled for multiple confounders [34]. A p-value ≤ 0.05 was considered statistically significant. All analyzes were performed using IBM SPSS Statistics version 28.0.

## Results

### Descriptive results

**Fig 1:**
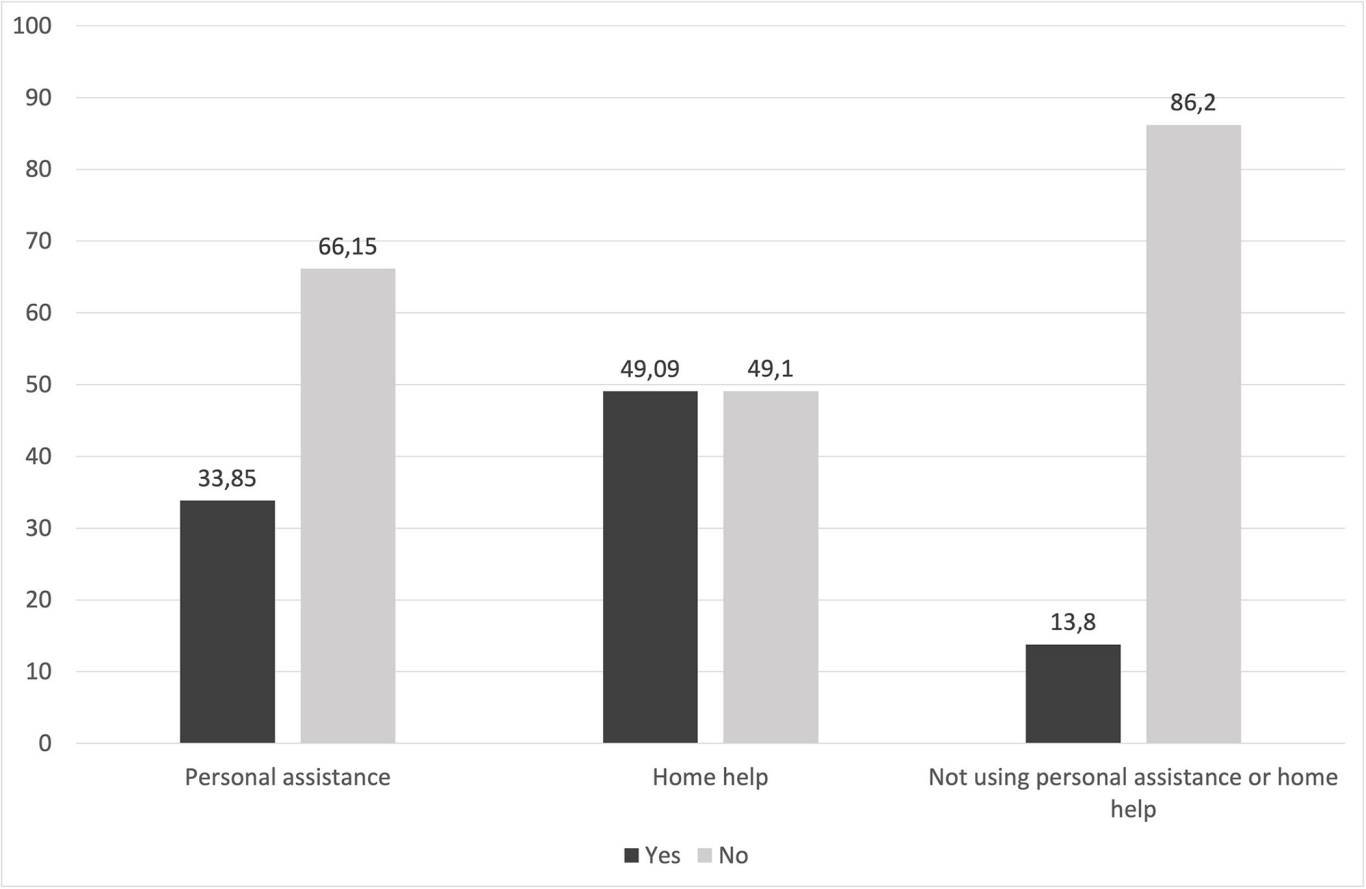
Distribution of the use of informal help among categories of persons with multiple sclerosis.

Sample characteristics are presented in Table 1. Fifteen of the respondents used both personal assistance and home help. As is evident, a large majority of respondents were not using formal help. Among the groups of persons with MS, the distributions of sex and type of residential area were similar. There were several variables for which persons who used formal help differed from those who did not. Persons with MS who used formal help were generally older, less educated, born outside the Nordic countries, living alone, receiving sickness benefits, had less disposable income, and received informal help to a larger extent. The latter was especially evident for those who used home help (see Fig 1).

**Table 1:** Descriptive statistics of responses from 3,951 persons with MS asked if they received formal help. The categories of personal assistance and home help are not mutually exclusive, and 15 respondents used both. Due to the small number of observations regarding age and EDSS score, outcomes were merged to protect the identities of respondents.

	Personal assistance (PA)	Home help (HH)	Not using PA or HH
	n = 66	n = 57	n = 3843
**Sex** n (%)			
Female	42 (63.6)	36 (63.2)	2768 (72)
Male	24 (36.4)	21 (36.8)	1075 (28)
**Age** n (%)			
20–40 years	13 (19.7)	16 (28.07)	1821 (47.47)
41–51 years	53 (80.3)	41 (71.93)	2015 (52.53)
mean (SD)	44.3 (4.99)	42.1 (6.60)	39.9 (7.22)
**Education level (years in school)** n (%)			
0–12	38 (57.6)	31 (54.4)	1482 (38.6)
12 <	28 (42.4)	26 (45.6)	2361 (61.4)
**Country of birth** n (%)			
Nordic countries	52 (78.8)	48 (84.2)	3449 (89.84)
Outside Nordic countries	14 (21.2)	9 (15.8)	390 (10.16)
**Cohabitation** n (%)			
Living alone	31 (47)	24 (42.1)	537 (13.98)
Cohabiting with adult and/or child(ren)	35 (53)	33 (57.9)	3304 (86.02)
**Receiving sickness benefit** n (%)			
Yes	47 (72.31)	38 (66.7)	737 (19.23)
No	18 (27.69)	19 (33.3)	3096 (80.77)
**Type of residential area** n (%)			
City	28 (42.4)	29 (50.9)	1710 (44.57)
Town/suburb	28 (42.4)	19 (33.3)	1480 (38.57)
Rural area	10 (15.2)	9 (15.8)	647 (16.86)
**Disposable income 2019** n (%)			
< SEK 165 240/year	34 (51.5)	27 (47.4)	623 (16.25)
> SEK 165 240/year	32 (48.5)	30 (52.6)	3212 (83.75)
mean (SD)	215 294 (180 062)	220 712 (165 459)	301 047 (171 934)
**Receiving informal help** n (%)			
Yes	22 (33.85)	27 (49.09)	527 (13.8)
No	43 (66.15)	28 (49.1)	3298 (86.2)
**Most limiting symptom of MS** n (%)			
Invisible symptom	25 (43.1)	35 (62.5)	2438 (68.46)
Visible symptom	32 (55.17)	20 (35.71)	649 (18.23)
**EDSS** n (%)			
0–5.5	13 (27.08)	21 (47.73)	2972 (96.62)
6–9.5	35 (72.92)	23 (52.27)	104 (3.38)
**Presence of another long-term disease/impairment** n (%)			
Yes	18 (27.3)	24 (44.44)	1190 (31.27)
No	48 (72.7)	30 (55.56)	2616 (68.73)

SD–Standard Deviation

SEK–Swedish Krona

**Fig 2:**
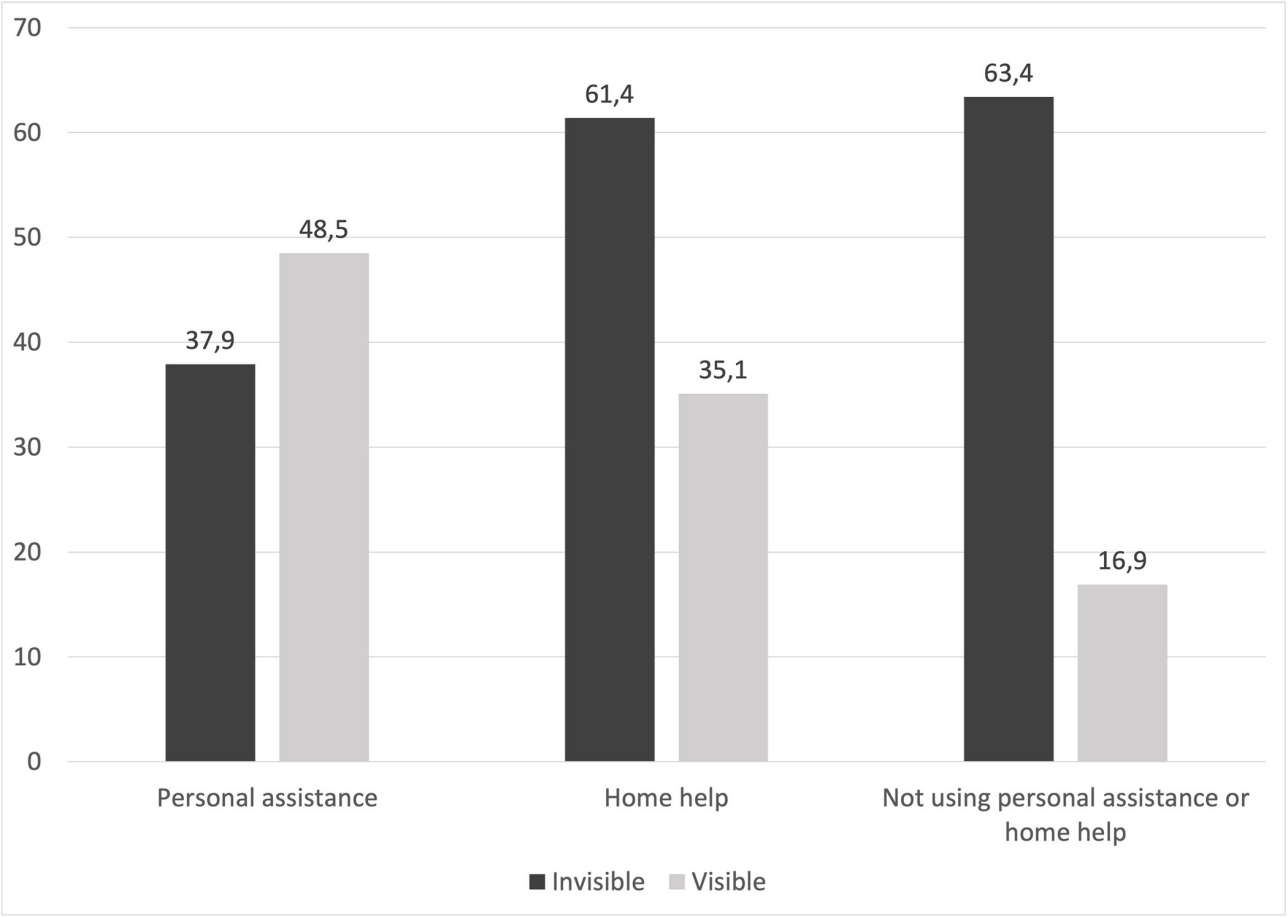
Distribution of most limiting symptom stated among categories of persons with multiple sclerosis.

The use of informal help was not the only variable for which differences between persons with MS using personal assistance and those using home help were found. Both groups using formal help had substantially higher EDSS scores than those not using formal help. However, the proportion of persons with the highest scores was greater among those using personal assistance than those using home help. Another difference between the groups was the presence of another long-term disease or impairment. The proportion that had another form of long-term disease or impairment was clearly larger among those using home help than those using personal assistance. The variable for most limiting symptom of MS revealed a distinct difference as well (see Fig 2). The proportion of persons who stated an invisible symptom as being the most limiting was approximately the same for those using home help and those not using any formal help. Among these groups, a large majority responded that their most limiting symptom was invisible. In contrast, the majority of persons with MS using personal assistance stated a visible symptom as being the most limiting.

### Results from logistic regression analysis

Unadjusted odds ratios for using personal assistance are presented for comparing changes when entered into the model (Table 2). In the mutually adjusted logistic regression model, five of 12 variables remained statistically significant. The most tangible association with the use of personal assistance was an EDSS score between 6 and 9.5 (adjusted OR 18.83, 95% CI 6.94–51.13). Four other variables were found to have a statistically significantly association with the use of personal assistance: living alone, receiving sickness benefit, a visible symptom as the most limiting one, and a disposable income below the limit of risk of poverty. They all had smaller odds (adjusted OR ranging from 3.32 to 2.16). Most of the variables were not significantly associated with usage of personal assistance in the adjusted model.

**Table 2:** Unadjusted and mutually adjusted odds ratios from binary logistic regression analyses for characteristics associated with the use of personal assistance among persons with multiple sclerosis, n = 3,863.

				Unadjusted			Adjusted		
			OR		95% CI			OR	95% CI
**Sex**									
male			1.46		0.88–2.42			0.88	0.46–1.67
(ref = female)									
**Age**									
31–40			4.03		0.52–31.09			3.37	0.40–28.56
41–51			11.95*		1.65–86.62			6.76	0.83–54.87
(ref = 20–30)									
**Education level (years in school)**									
0–12 years			2.14**		1.31–3.50			0.99	0.54–1.79
(ref = >12 years)									
**Country of birth**									
Outside Nordic countries			2.37**		1.30–4.31			1.73	0.79–3.78
(ref = Nordic countries, incl. Sweden)									
**Type of residential area**									
Towns and suburbs			1.16		0.68–1.97			1.07	0.56–2.05
Rural areas			0.95		0.46–1.96			0.83	0.35–1.96
(ref = cities)									
**Cohabitation**									
Living alone			5.29***		3.23–8.65			3.32***	1.79–6.15
(ref = cohabitating)									
**Disposable income**									
< SEK 165 240/year			5.31***		3.25–8.67			2.16*	1,13–4.13
(ref = > SEK 165 240/year)									
**Receiving sickness benefits**									
yes			10.51***		6.07–18.20			3.32**	1.62–6.81
(ref = no)									
**Receiving informal help**									
yes			3.07***		1.82–5.17			0.67	0.35–1.24
(ref = no)									
**EDSS**									
3–5.5			3.53*		1.15–10.84			1.81	0.54–6.03
6–9.5			88.06***		40.02–193.76			18.83***	6.94–51.13
(ref = 0–2.5)									
**Presence of another long-term disease/impairment**									
yes			0.82		0.47–1.41			0.57	0.31–1.07
(ref = no)									
**Most limiting symptom**									
visible symptom			4.72***		2.78–8.03			2.73**	1.47–5.06
no symptom			0.21		0.03–1.54			0.51	0.06–4.06
(ref = invisible symptom)									

Model: Nagelkerke R Square 0.378, Hosmer and Lemeshow Test 0.909

Note: OR = Odds Ratio; CI = Confidence Interval

***p < 0.001

**p < 0.01

*p < 0.05

Unadjusted odds ratios for the use of home help are presented for comparing changes when entered into the full model (Table 3). In the mutually adjusted logistic regression model, four of 12 variables remained statistically significant. The strongest association with the use of home help was an EDSS score between 6 and 9.5 (adjusted OR 6.83, 95% CI 2.67–17.46). The remaining significant variables were living alone, receiving sickness benefit, and receiving informal help (adjusted OR ranging from 2.56 to 1.89). Most variables were not significantly associated with the use of home help in the adjusted model.

**Table 3:** Unadjusted and mutually adjusted odds ratios from binary logistic regression analyses for characteristics associated with the use of home help among persons with multiple sclerosis, n = 3,863.

				Unadjusted			Adjusted		
			OR		95% CI			OR	95% CI
**Sex**									
male			1.49		0.87–2.57			1.14	0.60–2.19
(ref = female)									
**Age**									
31–40			0.73		0.25–2.12			0.58	0.19–1.77
41–51			1.82		0.72–4.64			1.03	0.37–2.91
(ref = 20–30)									
**Education level (years in school)**									
0–12 years			1.87*		1.11–3.17			1.19	0.64–2.20
(ref = >12 years)									
**Country of birth**									
Outside Nordic countries			1.63		0.79–3.35			1.09	0.46–2.58
(ref = Nordic countries, incl. Sweden)									
**Type of residential area**									
Towns and suburbs			0.75		0.42–1.35			0.77	0.40–1.50
Rural areas			0.82		0.39–1.74			0.84	0.36–1.94
(ref = cities)									
**Cohabitation**									
Living alone			4.29***		2.52–7.31			2.56**	1.36–4.82
(ref = cohabitating)									
**Disposable income**									
< SEK 165 240/year			4.46***		2.63–7.54			1.69	0.89–3.24
(ref = > SEK 165 240 /year)									
**Receiving sickness benefit**									
yes			7.96***		4.56–13.88			2.56*	1.24–5.31
(ref = no)									
**Receiving informal help**									
yes			5.87***		3.43-10-03			1.89*	1.00–3.56
(ref = no)									
**EDSS**									
3–5.5			2.83*		1.136–7.051			1.56	0.57–4.21
6–9.5			30.12***		15.17–59.82			6.83***	2.67–17.46
(ref = 0–2.5)									
**Presence of another long-term disease/impairment**									
yes			1.76*		1.03–3.03			1.30	0.72–2.35
(ref = no)									
**Most limiting symptom**									
visible symptom			2.06*		1.18–3.60			1.24	0.65–2.37
no symptom			0.15		0.020–1.08			0.43	0.06–3.29
(ref = invisible symptom)									

Model: Nagelkerke R Square 0.235, Hosmer and Lemeshow Test 0.051

Note: OR = Odds Ratio; CI = Confidence Interval

***p < 0.001

**p < 0.01

*p < 0.05

## Discussion

To the best of our knowledge, this is the first study examining the use of personal assistance and home help among Swedish persons with MS in relation to various background characteristics. Previous studies have investigated the use of several types of services/help, mainly with a focus on health care or sick leave [16, 24–26]. In contrast to the present article, previous studies have often included either older persons with MS [10, 20] or persons more severely affected with MS [18, 22].

The central finding of this study is that the single most important variable associated with the use of formal help (e.g., personal assistance and home help) is the EDSS score. In other words, the degree of impairment seems to be the determining factor in who receives certain forms of formal help. Several demographic variables were controlled for in the models but were found to not be significantly associated with the use of formal help. The full models indicate no significant differences that could be attributed to unequal distribution due to demographic characteristics such as sex, country of birth, type of residential area, or education level. Instead, the results obtained reflect the intention of the universal welfare model, by which personal assistance and home help are supposed to be granted to persons in need of help due to impairment and/or old age. It is not surprising that there was a statistically significant association between receiving sickness benefit and a higher EDSS score, as a higher degree of impairment would be expected to increase the probability of someone receiving sickness benefits [25].

Living alone was a statistically significant factor in predicting the use of both personal assistance and home help. This result is consistent with previous research, which has shown that persons with MS often receive informal help from a partner or other family member [14, 16, 18, 21]. However, this can be interpreted in different ways. One plausible explanation might be that persons with MS in need of help primarily ask family, avoiding interference from external paid employees out of a sense of personal integrity. Another possible explanation might be that those who cohabit are more likely to receive informal help from the person(s) with whom they live, which serves as a necessary complement to inadequate formal help [35]. From the total sample, it was evident that the use of informal help was significantly more common than formal help, which is in line with previous research from various national contexts [14, 16–18].

A disposable income below the poverty-risk level was found to be associated with use of personal assistance. This might be explained, at least partly, by the fact that persons with MS who use personal assistance in general are more severely impaired, and consequently often not in paid work [25]. However, this can also be seen as indicative of the austerity measures applied in Sweden over recent years [7]. Descriptive statistics show that about half of those using formal help have a disposable income below the poverty-risk level, leaving them particularly vulnerable.

In relation to the open question about the most limiting symptom of MS, there was a statistically significant association between visible symptoms and the use of personal assistance, while the association was nonsignificant in relation to home help. As was found at the descriptive level, there was a clear distinction between the number of persons with MS mainly affected by an invisible symptom using personal assistance compared to home help. Persons with MS who used home help more often rated an invisible symptom as being the most limiting. This was also the case for the group that did not use any formal help. As has been found previously, persons with MS mainly affected by invisible symptoms may be assessed as being less severely impacted by the disease [4] and encounter difficulties obtaining formal help [26]. The results of this study support these findings. Rating a visible symptom of MS as being the most limiting increased the probability of a person receiving a more comprehensive form of formal help, while an invisible symptom increased the likelihood of them receiving a less comprehensive form of formal help, as well as receiving informal help. Thus, there seems to be a difference in how persons with MS are assessed and furthermore in what formal help they may receive, due to the character of their symptoms. The results of the present study may indicate unequal treatment. The results might also imply an interplay in which the presence of informal help compensates for additional help that would otherwise be needed and is not available within the framework of home help [15, 19].

Future research may improve the state of knowledge in this field in several ways. In contrast to this cross-sectional study, a longitudinal study could detect variables associated with the use of formal help over time. Like previous research from other national contexts, comparisons in the use of formal help between persons with different diseases/impairments may reveal important differences. Qualitative research on persons with MS and the use of formal help would deepen the understanding of the unique experiences of individuals. Studies focusing on informal help in Sweden would also add to the limited existing knowledge. Lastly, it is suggested that future research further may explore the impact of invisible symptoms of MS, both in itself, but also in relation to formal and informal help. The possible interplay between invisible symptoms and informal help is another topic that future research might explore further.

The findings of the present article may provide guidance for MS care to improve. In the health care, physicians and other professionals need to address the topic on what symptoms mainly affects persons with MS without relying primarily on what is observable. Invisible symptoms have been found to be highly prevalent for persons with MS [4], which is supported by this study as well. If health care professionals can identify how persons with MS are affected by the disease, they may support these persons in a more holistic way. This includes support from relevant health care professionals in coping with the disease and symptoms, but also better support when persons with MS apply for formal help. These recommendations also involve other professionals in the public sector that encounters persons with MS, for example, social workers.

This study contributes new knowledge. The combination of data from three sources provides rich information on every respondent, and it enabled a comparison of the use of formal help in relation to many socioeconomic background variables. The large sample includes a considerable proportion of persons with MS in Sweden, and studies on younger persons with MS and formal help are scarce. However, it is important to acknowledge that there are limitations to the present study. Firstly, it is not known if those who did not respond to the survey constitute a specific subgroup of persons with MS. It might be assumed that non-respondents are more likely to belong to groups with limited knowledge of Swedish [36] or who are more severely affected by MS and/or other diseases [37]. The sample included persons with MS between 20 and 51 years old, focusing on those of younger/working age. A sample that also included persons with MS in older age groups would have provided a better overall picture of the population. It should be remembered that persons aged 65 or older in Sweden cannot apply for personal assistance. However, those who are already receiving personal assistance prior to the age of 65 can continue to receive it beyond that age. The variable for receiving sickness benefit was recoded as a binary variable. The original question on occupational status given to respondents allowed for more than one response. While the recoded variable did not accommodate this complex picture of respondents’ situations, it served the primary interest of identifying those who received some form or level of sickness benefit. Furthermore, there may be factors not measured in the survey that may have influenced the relationship, e.g., residual confounding.

## Conclusions

This study on formal help among persons with MS in Sweden found that degree of impairment, determined by EDSS score, was the factor most closely associated with the use of personal assistance and home help. Two other factors were also both associated with the use of personal assistance and home help: living alone and receiving sickness benefits. The use of personal assistance was associated with a visible symptom being the most limiting and having a disposable income below the poverty-risk limit. Home help was associated with receiving informal help. Several background factors were controlled for but were not related to differences in the use of these forms of formal help. The results indicate that the intention of the universal welfare model, that formal help is received based on individual need due to impairment and/or old age, is being fulfilled. However, differences between users of personal assistance and home help were found at the descriptive level. Persons with MS using home help were considerably more likely to be mainly affected by an invisible symptom and use informal help.

## Supporting information

S1 Table Full mutually adjusted binary logistic regression model for characteristics associated with the use of personal assistance among persons with multiple sclerosis, n = 3,863. (DOCX)

S2 Table Full mutually adjusted binary logistic regression model for characteristics associated with the use of home help among persons with multiple sclerosis, n = 3,863. (DOCX)

## References

[1] The Multiple Sclerosis International Federation. Atlas of MS, 3rd edition. 2020.

[2] Ahlgren C, Odén A, Lycke J. High nationwide prevalence of multiple sclerosis in Sweden. Multiple sclerosis. 2011;17(8):901–8. doi: 10.1177/1352458511403794 21459810

[3] Filippi M, Piehl F, Preziosa P, Solari A, Vukusic S, Rocca M. Multiple sclerosis (Primer). Nature Reviews: Disease Primers. 2018;4(1). doi: 10.1038/s41572-018-0041-4 30410033

[4] Gustavsen S, Olsson A, Søndergaard HB, Andresen SR, Sørensen PS, Sellebjerg F, et al. The association of selected multiple sclerosis symptoms with disability and quality of life: a large Danish self-report survey. BMC Neurology. 2021;21(1). doi: 10.1186/s12883-021-02344-z 34399707 PMC8365982

[5] World Health Organization. World Report on Disability. 2011. 26131540

[6] Altermark N, Nilsson H. State measurements of benefit fraud: Why expert elicitations cannot be used to measure incorrect personal assistance payments. Scandinavian journal of disability research: SJDR. 2020;22(1):158–67. doi: 10.16993/SJDR.610

[7] Järkestig Berggren U, Melin Emilsson U, Bergman A-S. Strategies of austerity used in needs assessments for personal assistance–changing Swedish social policy for persons with disabilities. European Journal of Social Work. 2021;24(3):380–92. doi: 10.1080/13691457.2019.1639627

[8] von Granitz H, Sonnander K, Reine I, Winblad U. Do personal assistance activities promote participation in society for persons with disabilities in Sweden? A five-year longitudinal study. Disability and rehabilitation. 2021:1–9. doi: 10.1080/09638288.2021.1897691 33721545

[9] Hughes N, Locock L, Ziebland S. Personal identity and the role of ‘carer’ among relatives and friends of people with multiple sclerosis. Social science & medicine (1982). 2013;96:78–85. doi: 10.1016/j.socscimed.2013.07.023 24034954 PMC3778435

[10] Zhang W, Sun H. Formal and informal care received by middle-aged and older adults with chronic conditions in Canada: CLSA data. PloS one. 2020;15(7):e0235774–e. doi: 10.1371/journal.pone.0235774 32634161 PMC7340302

[11] The National Board of Health and Welfare. Statistics on Care and Services for Persons with Impairments 2021. 2022 2022-4-7811 1.

[12] SFS. The Social Services Act (SSA). Swedish Code of Statutes 2001:453.; 2001:453.

[13] SFS. Act on Support and Service for Persons with Certain Functional Impairments (LSS). Swedish Code of Statutes 1993:387.; 1993:387.

[14] Mitchell LA, Hirdes J, Poss JW, Slegers-Boyd C, Caldarelli H, Martin L. Informal caregivers of clients with neurological conditions: profiles, patterns and risk factors for distress from a home care prevalence study. BMC health services research. 2015;15(1):350–. doi: 10.1186/s12913-015-1010-1 26315104 PMC4552273

[15] Patten SB, Williams JVA, Lavorato DH, Terriff D, Metz LM, Berzins S, et al. Perceived met and unmet health-care needs in a community population with multiple sclerosis. International journal of MS care. 2012;14(1):2–8. doi: 10.7224/1537-2073-14.1.2 24453726 PMC3882973

[16] Gottberg K, Einarsson U, Ytterberg C, Fredrikson S, von Koch L, Holmqvist LW. Use of health care services and satisfaction with care in people with multiple sclerosis in Stockholm County: A population-based study. Multiple sclerosis. 2008;14(7):962–71. doi: 10.1177/1352458508089688 18573818

[17] O’Hara L, De Souza L, Ide L. The nature of care giving in a community sample of people with multiple sclerosis. Disability and rehabilitation. 2004;26(24):1401–10. doi: 10.1080/09638280400007802 15764360

[18] Buchanan RJ, Radin D, Chakravorty BJ, Tyry T. Informal care giving to more disabled people with multiple sclerosis. Disability and rehabilitation. 2009;31(15):1244–56. doi: 10.1080/09638280802532779 19802928

[19] Freeman JA, Thompson AJ. Community services in multiple sclerosis: still a matter of chance. Journal of neurology, neurosurgery and psychiatry. 2000;69(6):728–32. doi: 10.1136/jnnp.69.6.728 11080223 PMC1737174

[20] Putnam M, Tang F. Multiple sclerosis, aging and support service utilization. The Journal of rehabilitation. 2007;73(4):3–14.

[21] McCrone P, Heslin M, Knapp M, Bull P, Thompson A. Multiple Sclerosis in the UK: Service Use, Costs, Quality of Life and Disability. PharmacoEconomics. 2008;26(10):847–60. doi: 10.2165/00019053-200826100-00005 18793032

[22] Borreani C, Bianchi E, Pietrolongo E, Rossi M, Cilia S, Giuntoli M, et al. Unmet needs of people with severe multiple sclerosis and their carers: Qualitative findings for a home-based intervention. PloS one. 2014;9(10):e109679-e. doi: 10.1371/journal.pone.0109679 25286321 PMC4186842

[23] Roddam H, Rog D, Janssen J, Wilson N, Cross L, Olajide O, et al. Inequalities in access to health and social care among adults with multiple sclerosis: A scoping review of the literature. Multiple sclerosis and related disorders. 2019;28:290–304. doi: 10.1016/j.msard.2018.12.043 30641354

[24] Ytterberg C, Johansson S, Gottberg K, Holmqvist L, von Koch L. Perceived needs and satisfaction with care in people with multiple sclerosis: A two-year prospective study. BMC neurology. 2008;8(1):36–. doi: 10.1186/1471-2377-8-36 18823543 PMC2569963

[25] Sundström P, Nyström L, Svenningsson A, Forsgren L. Sick leave and professional assistance for multiple sclerosis individuals in Västerbotten County, northern Sweden. Multiple sclerosis. 2003;9(5):515–20. doi: 10.1191/1352458503ms955oa 14582779

[26] Johansson S, Ytterberg C, Gottberg K, Widén Holmqvist L, von Koch L. Use of health services in people with multiple sclerosis with and without fatigue. Multiple sclerosis. 2009;15(1):88–95. doi: 10.1177/1352458508095730 18701570

[27] Hillert J, Stawiarz L. The Swedish MS registry—clinical support tool and scientific resource. Acta Neurol Scand 2015: 132 (Suppl. 199): 11–19. © 2015 John Wiley & Sons A/S. Published by John Wiley & Sons Ltd. doi: 10.1111/ane.12425 PMC4657484 26046553

[28] Ludvigsson JF, Svedberg P, Olén O, Bruze G, Neovius M. The longitudinal integrated database for health insurance and labour market studies (LISA) and its use in medical research. European journal of epidemiology. 2019;34(4):423–37. doi: 10.1007/s10654-019-00511-8 30929112 PMC6451717

[29] Eurostat. Eurostat Regional Yearbook: 2021 Edition. Union POotE; 2021 2022-06-07. Report No.

[30] National Institute for Health and Care Excellence. Multiple sclerosis in adults: management. Clinical guideline National Institute for Health and Care Excellence MS symptom management and rehabilitation 2014. p. 12–7.

[31] National MS Society. 2020. Available from: https://www.nationalmssociety.org/Research/Research-News-Progress.

[32] Kurtzke JF. Rating neurologic impairment in multiple sclerosis: an expanded disability status scale (EDSS). Neurology. 1983;33(11):1444–52. doi: 10.1212/wnl.33.11.1444 6685237

[33] Hosmer DW, Lemeshow S, Sturdivant RX. Applied logistic regression. 3rd ed. ed. Chicester: Chicester: Wiley; 2013.

[34] Pourhoseingholi MA, Baghestani AR, Vahedi M. How to control confounding effects by statistical analysis. Gastroenterology and hepatology from bed to bench. 2012;5(2):79–83. 24834204 PMC4017459

[35] Dunér A, Olin E. Personal assistance from family members as an unwanted situation, an optimal solution or an additional good? The Swedish example. Disability & society. 2018;33(1):1–19. doi: 10.1080/09687599.2017.1375900

[36] Ahlmark N, Algren MH, Holmberg T, Norredam ML, Nielsen SS, Blom AB, et al. Survey nonresponse among ethnic minorities in a national health survey—a mixed-method study of participation, barriers, and potentials. Ethnicity & health. 2015;20(6):611–32. doi: 10.1080/13557858.2014.979768 25411892

[37] Fry A, Littlejohns TJ, Sudlow C, Doherty N, Adamska L, Sprosen T, et al. Comparison of Sociodemographic and Health-Related Characteristics of UK Biobank Participants With Those of the General Population. American journal of epidemiology. 2017;186(9):1026–34. doi: 10.1093/aje/kwx246 28641372 PMC5860371

