# Supplementary material for: Formal help for persons with multiple sclerosis—Background factors associated with usage of personal assistance and home help in Sweden
Source: PLoS One. 2023 May 18;18(5):e0286010. doi: 10.1371/journal.pone.0286010 (PMC10194947; doi:10.1371/journal.pone.0286010)
Supplement: S1 Table — (DOCX) [file pone.0286010.s001.docx]

**Table S1**

|  | B | SE | Wald | df | p | OR | 95% CI |
| --- | --- | --- | --- | --- | --- | --- | --- |
| Sex  male  (ref = female) | -0.130 | 0.327 | 0.157 | 1 | 0.692 | 0.878 | 0.462-1.668 |
| Age  31-40  41-51  (ref = 20-30) | 1.216  1.912 | 1.090  1.068 | 1.245  3.203 | 1  1 | 0.264  0.073 | 3.374  6.764 | 0.399-28.560  0.834-54.871 |
| Education level (years in school)  0-12 years  (ref = >12 years) | -0.013 | 0.305 | 0.002 | 1 | 0.966 | 0.987 | 0.543-1.795 |
| Country of birth  Outside Nordic countries  (ref = Nordic countries, incl. Sweden) | 0.547 | 0.399 | 1.877 | 1 | 0.171 | 1.727 | 0.790-3.776 |
| Type of residential area  Towns and suburbs  Rural areas  (ref = cities) | 0.072  -0.190 | 0.329  0.439 | 0.048  0.188 | 1  1 | 0.826  0.665 | 1.075  0.827 | 0.564-2.050  0.349-1.956 |
| Cohabitation  Living alone  (ref = cohabitating) | 1.200 | 0.315 | 14.480 | 1 | < 0.001 | 3.318 | 1.789-6.155 |
| Disposable income  < SEK 165 240/year  (ref = > SEK 165 240/year) | 0.771 | 0.330 | 5.447 | 1 | 0.020 | 2.161 | 1.131-4.128 |
| Receiving sickness benefits  yes  (ref = no) | 1.199 | 0.367 | 10.706 | 1 | 0.001 | 3.318 | 1.618-6.807 |
| Receiving informal help  yes  (ref = no) | -0.407 | 0.335 | 1.479 | 1 | 0.224 | 0.666 | 0.346-1.283 |
| EDSS  3-5.5  6-9.5  (ref = 0-2.5) | 0.595  2.936 | 0.613  0.510 | 0.940  33.187 | 1  1 | 0.332  < 0.001 | 1.813  18.832 | 0.545-6.031  6.937-51.127 |
| Presence of another long-term disease/impairment  yes  (ref = no) | -0.557 | 0.321 | 3.006 | 1 | 0.083 | 0.573 | 0.306-1-075 |
| Most limiting symptom  visible symptom  no symptom  (ref = invisible symptom) | 1.005  -0.664 | 0.314  1.054 | 10.214  0.397 | 1  1 | 0.001  0.528 | 2.732  0.515 | 1.475-5.060  0.065-4.060 |
| Constant | -8.101 | 1.137 | 50.756 | 1 | < 0.001 | 0.000 |  |
