# Supplementary material for: Formal help for persons with multiple sclerosis—Background factors associated with usage of personal assistance and home help in Sweden
Source: PLoS One. 2023 May 18;18(5):e0286010. doi: 10.1371/journal.pone.0286010 (PMC10194947; doi:10.1371/journal.pone.0286010)
Supplement: S2 Table — (DOCX) [file pone.0286010.s002.docx]

**Table S2**

|  | B | SE | Wald | df | p | OR | 95% CI |
| --- | --- | --- | --- | --- | --- | --- | --- |
| Sex  male  (ref = female) | 0.134 | 0.332 | 0.163 | 1 | 0.686 | 1.143 | 0.597-2.190 |
| Age  31-40  41-51  (ref = 20-30) | -0.552  0.032 | 0.574  0.529 | 0.923  0.004 | 1  1 | 0.337  0.952 | 0.576  1.032 | 0.187-1.775  0.366-2.914 |
| Education level (years in school)  0-12 years  (ref = >12 years) | 0.171 | 0.315 | 0.293 | 1 | 0.588 | 1.186 | 0.640-2.200 |
| Country of birth  Outside Nordic countries  (ref = Nordic countries, incl. Sweden) | 0.090 | 0.437 | 0.042 | 1 | 0.837 | 1.094 | 0.465-2.577 |
| Type of residential area  Towns and suburbs  Rural areas  (ref = cities) | -0.257  -0.171 | 0.339  0.426 | 0.577  0.160 | 1  1 | 0.447  0.689 | 0.773  0.843 | 0.398-1.502  0.365-1.945 |
| Cohabitation  Living alone  (ref = cohabitating) | 0.940 | 0.322 | 8.513 | 1 | 0.004 | 2.561 | 1.362-4.816 |
| Disposable income  < SEK 165 240/year  (ref = > SEK 165 240/year) | 0.527 | 0.331 | 2.539 | 1 | 0.111 | 1.694 | 0.886-3.241 |
| Receiving sickness benefits  yes  (ref = no) | 0.942 | 0.372 | 6.418 | 1 | 0.011 | 2.565 | 1.238-5.315 |
| Receiving informal help  yes  (ref = no) | 0.637 | 0.323 | 3.878 | 1 | 0.049 | 1.890 | 1.003-3.563 |
| EDSS  3-5.5  6-9.5  (ref = 0-2.5) | 0.442  1.922 | 0.508  0.479 | 0.755  16.114 | 1  1 | 0.385  < 0.001 | 1.556  6.832 | 0.574-4.214  2.674-17.460 |
| Presence of another long-term disease/impairment  yes  (ref = no) | 0.264 | 0.301 | 0.772 | 1 | 0.380 | 1.302 | 0.722-2.348 |
| Most limiting symptom  visible symptom  no symptom  (ref = invisible symptom) | 0.215  -0.850 | 0.331  1.041 | 0.422  0.666 | 1  1 | 0.516  0.414 | 1.240  0.428 | 0.649-2.369  0.056-3.289 |
| Constant | -5.786 | 0.596 | 94.340 | 1 | < 0.001 | 0.003 |  |
